# A Language Model for Pediatric Occupational Therapy Documentation: Model Development and Pilot Study

**DOI:** 10.2196/73274

**Published:** 2026-05-15

**Authors:** Rachel DiMaio, Tia Tuinstra, Trevor Yu, Ilona Koshy, Brendan Wylie-Toal, Bryan Tripp

**Affiliations:** 1 Department of Systems Design Engineering, Faculty of Engineering, University of Waterloo, 200 University Ave W, Waterloo, N2L 3G1, Canada, 1 (519) 888-4567; 2 KidsAbility Centre for Child Development, Waterloo, ON, Canada; 3 School of Environment, Enterprise and Development, Faculty of the Environment, University of Waterloo, Waterloo, ON, Canada

**Keywords:** SOAP notes, large language models, occupational therapy, documentation, model development, electronic medical records, natural language processing, pilot study

## Abstract

**Background:**

In occupational therapy, progress notes and other client-related administrative tasks are essential for providing treatment but are time-consuming. Therapists spend at least as much time on these tasks as providing care, which contributes to growing waitlists.

**Objective:**

This study aimed to create a custom large language model to make the process of writing progress notes more efficient by converting point-form scratch notes from pediatric occupational therapy treatment sessions into draft documentation in subjective-objective-assessment-plan format.

**Methods:**

Using a dataset of redacted historical progress notes, various training methods, including domain-adaptive pretraining and low-rank adaptation fine-tuning, were applied to train Llama 2 and 3 models. Since the historical notes lacked corresponding scratch notes, few-shot prompting with human-in-the-loop evaluations was used to generate synthetic scratch notes. This pairing of historical notes and generated scratch notes enabled effective fine-tuning of the Llama models on the desired task. The final model, a fine-tuned Llama 3 8B Instruct model, was piloted in a pediatric rehabilitation center and compared with Microsoft Copilot. Ten therapists used both models for 3 weeks each.

**Results:**

The custom model notes scored higher than manually written notes on clarity, completeness, relevance, and organization (*P*<.001) , and similarly on conciseness. They scored higher than those from Copilot on conciseness (*P*<.001). However, in this small pilot, a significant reduction in time spent on documentation when using the custom model versus manual notes was not detected. Follow-up investigation revealed that time savings were observed only when therapists were coached to write sparse scratch notes; however, they tended to revert to detailed notes after coaching for which the model was not shown to improve efficiency.

**Conclusions:**

The model had the capacity to save time when therapists provided brief input to the model. However, in practice, therapists preferred to provide detailed input. Used in this way, the model improved note quality rather than saving time.

## Introduction

### Background

Clinical documentation is essential for continuity of care. Documentation has also become important for billing and legal protection in recent decades. These requirements have added complexity and volume, so that documentation requirements have come to compete with patient care and contribute to clinician burnout [1-5], which can lead to medical errors [6] and early retirement [7]. While physicians spend about half their time on documentation and administrative tasks [4,8,9], this burden is particularly acute in pediatric rehabilitation. Internal data from KidsAbility, the primary affiliation of some of the authors, suggest that therapists spend about 50% more time on client-related administrative work than interacting with clients. This creates barriers to access. For example, in our region, waitlists are growing longer as demand increases while therapist capacity is flat [10,11]. The process of completing documentation and other administrative tasks can also be repetitive and tedious for the occupational therapists, reducing job satisfaction [12]. The primary goal of this project was to develop a system that uses machine learning to reduce the amount of time that clinicians spend on documentation, with a focus on the needs of pediatric rehabilitation clinicians, including an emphasis on data privacy.

Machine learning systems are becoming widely adopted to reduce the burden of clinical documentation, performing tasks such as selecting billing codes and generating clinical reports and notes [13]. Many clinicians use automated scribe tools to create draft documentation from transcribed conversations with patients. These systems generate new text, summarize relevant findings, and/or retrieve information from existing sources. Some systems are also multimodal, using vision models to incorporate information from medical images [14]. Clinicians typically make amendments to these automated notes [15].

Biomedical large language models (LLMs) have the potential to reduce time spent by clinicians on documentation based on their success in medical language tasks [13]. Frontier models such as ChatGPT have been reported to perform well in these applications [16,17], and others have reported improvements with fine-tuned custom models [18]. The use of commercial models can raise questions about the privacy of health information because it requires sending patient data to artificial intelligence (AI) companies. Additionally, large generalist models have infrastructure requirements that can hinder their use in custom applications [19], and their routine use poses a threat to the environment [20]. Increases in model size are associated with higher computational, energy, and hardware demands during training and deployment, resulting in increased environmental impact. However, there are a number of smaller open-source LLMs with increasingly competitive performance in a variety of language tasks, which could potentially be fine-tuned to specialize in narrow clinical documentation tasks, performing them more efficiently, and under the control of the health care provider [21].

### Goal of This Study

The study explores the customization of a small LLM to write progress notes for occupational therapy appointments, a key documentation task in pediatric rehabilitation. Many therapists write progress notes based on point-form scratch notes taken during the appointment. The system was designed to convert these scratch notes into draft progress notes in the widely used subjective-objective-assessment-plan (SOAP) format [22]. The therapists would be required to review and edit each draft and would retain legal responsibility for their content. The research team developed and tested a system for this purpose in consultation with therapists at KidsAbility, a pediatric rehabilitation center in Southwestern Ontario. KidsAbility provides services for children and youth with communication, physical, or developmental needs, including occupational therapy (OT). A previously published abstract summarizes the key points of this research [23].

### Prior Work

Most of the previous work in clinical note generation has used visit transcripts as input [24-27]. This task is closely related to abstractive summarization and particularly to abstractive summarization of meetings [28]. In an early work, Enarvi et al [29] reported that transformers outperformed recurrent networks in this task. Krishna et al [25] compared several methods of generating SOAP notes from visit transcripts and found that it was helpful to separate the pipeline into multiple stages that first recognized and extracted salient utterances, then clustered these into groups of related utterances, and then generated a SOAP note sentence related to each cluster. Ramprasad et al [27], using transformer models, found that training different cross-attention parameters for each SOAP note section improved performance. The MEDIQA-Chat 2023 competition included the task of generating SOAP notes from patient-doctor conversations [30]. The winning entry [16] used few-shot inference with GPT-4, importantly selecting few-shot examples based on similarity with the input conversation. Biswas and Talukdar [17] also reported strong results using GPT-4 compared with several other models. Chen and Hirschberg [31] compared the summarization abilities of the 2 methods that performed best in MEDIQA-Chat (fine-tuning LLMs and GPTs) and showed that explicit prompting for SOAP style notes led to outputs with relevant information in all SOAP categories. More generally, LLMs such as ChatGPT have been shown to be effective in medical dialogue summarization, focusing on relevant medical facts [32].

At the time of writing, there are a number of commercial products that transcribe patient visits and generate various notes and reports, such as DeepScribe [33], Tali [34], Nuance Dragon Medical One [35], and AutoScribe [36]. Despite positive clinical reception, a recent study of one such tool found that editing was needed to maintain document quality, and that once this step was accounted for, the tool reduced documentation time by less than 10% [37]. Consistent with this, Knoll et al [15] reported that physicians felt that the value of such tools lay in allowing them to focus more on patients rather than in saving time.

Certain specifics of pediatric rehabilitation motivate the alternative approach of using scratch notes instead of visit transcripts as input. Pediatric rehabilitation transcripts are fragmented due to interaction between therapist, parent, and child. The visits tend to be longer than routine primary care visits (typically at least 45 minutes), and much of the conversation between therapists and children is only loosely related to the SOAP note. For example, some of therapists’ speech is meant to keep young children entertained. Furthermore, pediatric rehabilitation clients are typically less able to verbalize concerns due to factors including disability and the young age of clients, including preverbal clients. Overall, it is difficult to capture the treatment in dialogue. In contrast, physician SOAP notes often contain closely related (or even verbatim) [27] text from the transcript, such as the patient’s description of symptoms. Unspoken observations have been identified as a challenge [15], which may be even more prominent in pediatric rehabilitation. LLMs have a tendency to confabulate unknown details [38,39], and the fact that pediatric rehabilitation transcripts do not explicitly contain much of the necessary information may exacerbate this. Consistent with these challenges, therapists at KidsAbility reported mixed success in preliminary testing of a commercial transcription-based system. Finally, and most importantly, a large fraction of therapists work in public settings such as classrooms with many other students where recording and transcription are not possible. Scratch notes provide an input that is more focused, with less irrelevant information that might confuse the model, and that includes unspoken observations.

While this work was in review, there have been several related works published. For instance, Du et al [40] published a systematic review of the use of LLMs in electronic medical record (EMR) applications, finding 196 papers that used LLMs to analyze real-world EMR data. Notably, Du et al found that the majority (122/196, 62.2%) involved clinical decision support, with only 5.6% (11/196) of the studies related to text summarization tasks. Furthermore, the authors noted that many disciplines were not represented and that there is not yet a clinically meaningful evaluation framework that can be used for LLM applications. A more recent study that focuses on the summarization of EMR documentation is Dehkordi et al [41], which used a combination of highlights distinguishing detailed information in discharge notes and prompt engineering to improve the quality of summaries written by an LLM. Van Veen et al [42] showed that LLMs could outperform medical experts at the summarization of EMR data including progress notes, positing that LLMs could potentially be used in this capacity to help alleviate clinician documentation burden. However, a systematic review conducted by Bednarczyk et al [43] stresses that reliable performance assessments and clinical impact evaluations are largely absent from literature focusing on EMR summarization tasks. In the domain of clinical note generation using LLMs, there are several examples of frameworks developed to alleviate clinician documentation burden, including a framework for zero-shot and few-shot generation of emergency medical services documentation based on transcripts by Bai et al [44]; a pipeline to generate SOAP notes enhanced with keywords from doctor-patient conversations by Li et al [45]; SpecialtyScribe by Goyal et al [46], a set of modules for generating SOAP notes specific to a particular discipline based on transcripts; and a multimodal framework for generating SOAP notes based on skin lesion images and sparse clinical text by Kamal et al [47].

## Methods

**Figure 1.**
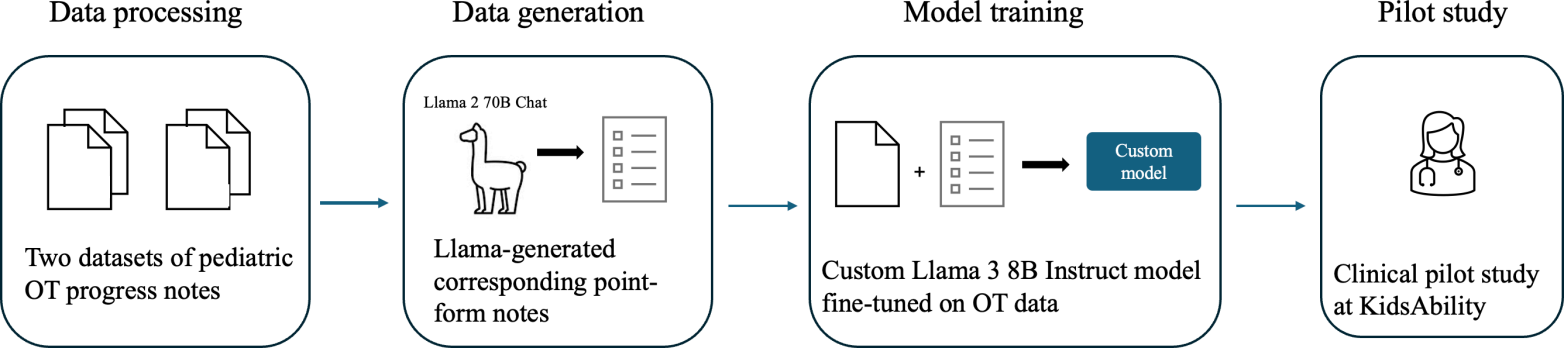
Our best-performing pipeline for custom language model development via fine-tuning Llama 3 8B Instruct. OT: occupational therapy.

The following subsections describe the data curation and model development processes. The model pipeline is summarized in Figure 1.

### Ethical Considerations

The use of historical EMR data for research purposes was approved by the University of Waterloo Research Ethics Board (institutional review board no. 45491). Specifically, the secondary use of these data was approved for research purposes without the requirement of additional informed consent. While personal health information (PHI) was not needed for the study and was explicitly excluded where possible by KidsAbility authorized professionals, the possibility of incidental PHI in the content of the notes used for training necessitated the approval of the ethics board. The families at KidsAbility provided general consent for the use of their data for research purposes that are Personal Health Information Protection Act–compliant. The parents were given the opportunity to restrict the use of their child’s data to not include research projects when they signed the KidsAbility Privacy policy. In the rare cases that this was requested, those data were excluded in this project. The data were made available in an environment on KidsAbility authorized servers that was compliant with the Personal Health Information Protection Act. All linked PHI was removed before the research team accessed the data, including all metadata concerning the client, such as their name, age, or location. Since LLMs can memorize portions of training data and potentially output them [48], any incidental PHI that appeared in the body of the text, such as first names, was also redacted. The redaction process was completed using a combination of the Amazon Comprehend service [49] and custom redaction code. First, the Comprehend service was set to automatically remove any names or ages from the text, replacing any found with the strings [NAME] and [AGE], respectively. The custom redaction code used an unlinked list of first names from KidsAbility to find and replace any remaining names.

### Datasets

#### Overview

To minimize editing after generation, the model was designed to produce draft SOAP notes in the same style as clinicians at KidsAbility. Ideal training data for this purpose would include historical SOAP notes linked to corresponding point-form scratch notes. However, because clinicians do not typically save their scratch notes, such paired data were not available.

KidsAbility had access to tens of thousands of historical SOAP notes stored in their previous EMR, GoldCare, which were migrated to their new system, AlayaCare, in 2023. The data were deidentified and made accessible to the research team via Amazon Web Services. Additionally, historical progress notes from AlayaCare were later provided to the research team within the same secure infrastructure.

#### Dataset Formatting

During data review, the research team found systematic differences between notes authored before and after the migration. Notes from AlayaCare, collected between January 2023 and March 2024, followed a more consistent SOAP structure, due to the use of standardized templates in the new EMR. In contrast, the older notes often lacked clear section headings and were highly variable in style. For clarity, these datasets are referred to as the GoldCare dataset (larger body of historical data from prior to the EMR migration) and the AlayaCare dataset (the smaller but better formatted dataset of notes from after the migration).

#### GoldCare Dataset

The GoldCare dataset consisted of 411,812 progress notes taken from 2012 to 2023. The data were filtered to include only OT progress notes, excluding speech-language therapy and physiotherapy notes. After this filtering, the dataset contained 37,437 OT notes.

The GoldCare dataset was also filtered by token length, using the Llama 2 7B tokenizer, to include notes with only 50-1000 tokens, accounting for the majority of the notes within the distribution. Inspection of examples showed that notes with less than 50 tokens were less likely to be proper SOAP notes, and a 1000-token maximum enabled faster, more cost-effective pretraining with a sequence length of 1024.

Furthermore, the dataset was divided into separate datasets including a domain-adaptive pretraining (DAPT) dataset (90%) and a fine-tuning dataset (10%). The division of the full dataset considered client IDs such that individual clients appeared only in one dataset or the other. Finally, the fine-tuning dataset was filtered once more to exclude any notes under 150 tokens since further inspection revealed that notes in the range of 50-150 tokens were less likely to conform to SOAP format. The pretraining dataset was not filtered further. This left 2298 notes in the fine-tuning dataset and 25,164 notes in the pretraining dataset.

#### AlayaCare Fine-Tuning Dataset

The AlayaCare dataset originally consisted of 1127 SOAP notes from OT appointments, which were used only for fine-tuning as the dataset was not large enough to support DAPT. This dataset was filtered to include notes with 100-1000 tokens, using the Llama 3 8B tokenizer, which is similar to the Llama 2 7B tokenizer. A smaller minimum length was allowed than in the GoldCare fine-tuning dataset because these notes were more reliably structured despite shorter token lengths. After filtering, the AlayaCare fine-tuning dataset comprised 973 notes in total.

### Scratch Note Generation

Achieving good performance with moderately sized LLMs often requires fine-tuning them on the task of interest [16]. For this purpose, examples of paired scratch notes (inputs) and SOAP notes (outputs) were necessary. However, the historical data did not contain scratch notes, so synthetic scratch notes corresponding to each SOAP note in the fine-tuning datasets were generated using few-shot inference with a Llama 2 70B model. A small set of high-quality examples illustrating the task (creating a scratch note based on a progress note) was provided in the prompt. The examples consisted of 45 scratch note and SOAP note pairs provided by clinicians at KidsAbility. Larger models such as Llama 2 70B excel in few-shot inference, mimicking the style and structure of provided examples [50], and were used here to capture the style and structure of KidsAbility scratch notes.

Past studies using LLMs to generate or augment training data [51-53] have found that it is important to optimize the quality of the generated data. Evaluation of factors such as realism, relevance, and biases can help ensure that the generated data are effective for fine-tuning. Accordingly, a human-in-the-loop evaluation process was used to find an effective generation approach that produced text of consistent quality when provided with a single example scratch note and SOAP note pair. A generation approach was iteratively refined, varying prompt formulations, sampling generation temperatures, and postprocessing steps. The effect of temperature was explored by varying the generation temperature within a predefined range (0.5‐0.9) while holding the other parameters constant, after which a temperature (0.8) was chosen based on qualitative assessment by the research team. The effect of different prompts and postprocessing steps was evaluated by clinician collaborators. Three OT clinicians scored scratch notes that were generated with different parameters and different few-shot examples in a 3-step evaluation process. The first step included 100 scratch notes, while the second and third included 80 each. In total, 7 different prompt formulations, 5 randomly selected few-shot example subsets, and 2 postprocessing techniques were assessed. To evaluate output stability, multiple scratch notes (n=4) were also generated with the same parameters.

The clinicians used a structured rubric to independently evaluate the scratch notes on realism, accuracy, and appropriate distinctions (between subjective reporting, goals, and observations). Each criterion was scored on a 5-point Likert scale (1 being the worst, and 5 being the best). An additional criterion, brevity, did not require clinical expertise and was therefore assessed by the research team. This criterion considered whether the model generated unwanted chat text such as, “Sure, here is a scratch note.” Additionally, the clinicians were asked to flag any notes that they considered particularly inaccurate or problematic and to provide qualitative feedback.

The scores were averaged across raters for each combination of prompt and postprocessing strategy. Descriptive statistics (mean and SD) together with qualitative feedback were used to select the final generation approach. The raters were generally concordant, but a formal ICC was not calculated. Using the selected parameters, the scratch notes were generated with all curated examples. For each generation, a few-shot example was selected programmatically, cycling through the available set to promote variability while preserving stylistic consistency.

### Model Training

#### Overview

The models chosen for training were the Llama 2 7B Chat model and then the Llama 3 8B Instruct model after its release in 2024. The smallest Llama models were chosen to minimize training and inference compute costs. Compared with other open-source LLMs, the Llama models are well documented, perform well on benchmarks, and performed best in our initial, exploratory testing. The Chat and Instruct versions of these models are those that have undergone fine-tuning and posttraining that refine the models’ ability to respond to chat prompts and instructions, which was desired for the given task.

For both DAPT and fine-tuning on the SOAP note task, the models were trained through causal language modeling (CLM), which is a self-supervised training process that tasks the model with autoregressively predicting the next token in a sequence [54]. The model was trained for a single epoch to reduce the risk of memorizing the training data and compromising privacy [48,55,56]. The hyperparameters were based on those used in the Llama 2 fine-tuning process [57]. Further implementation details, including the hyperparameters, can be found in Multimedia Appendix 1.

#### Domain-Adaptive Pretraining

LLMs are pretrained on massive text corpora. DAPT is a form of transfer learning that involves continued pretraining using CLM on text from a specific domain. DAPT is used to train a general pretrained model to understand domain-specific vocabulary and nuances and to produce text more suitable to the target domain [58]. The GoldCare pretraining dataset, composed of 25,164 historical progress notes, was used for DAPT. Training was performed for 1 epoch on the Llama 2 7B Chat model.

#### Fine-Tuning

In contrast with DAPT, fine-tuning is used to adapt a model to a particular task, rather than just a particular kind of text. Parameter-efficient fine-tuning was explored, specifically low-rank adaptation (LoRA), a prevalent method that trains large models efficiently in terms of both computation and the size of the fine-tuning dataset [59,60]. This method uses low-rank adapters, *n* × *n* weight matrices that consist of an outer product of 2 low-dimensional weight matrices (eg, *n* × 2 and 2 × *n*), forcing these weights to have low rank and few parameters. A low-rank weight matrix is summed with an existing *n* × *n* weight matrix. Only the low-rank adapters are optimized during LoRA fine-tuning while the original parallel weights are frozen.

The GoldCare and AlayaCare fine-tuning datasets were used for different fine-tuning training runs. Each of these datasets was further split into train and validation datasets, such that 80% of the data were in the train dataset and 20% were used for evaluation. Supervised fine-tuning was performed for a single epoch. The performance of these variations was evaluated as described in the “Qualitative Model Evaluation” section. The best-performing process, with a fine-tuned Llama 3 8B Instruct model, leading to a version that was piloted with clinicians, is shown in Figure 1.

### Qualitative Model Evaluation

There are no standard benchmarks for evaluating LLMs in SOAP note generation or related health care tasks [61]. For this reason, manual evaluations were performed to determine which model variation to pilot with therapists. These evaluations compared LoRA models vs fully fine-tuned models, models with vs without DAPT, Llama 2 vs Llama 3 models, and models trained on GoldCare vs AlayaCare data fine-tuning datasets. Additionally, the fine-tuned models were compared against Llama 2 Chat and Llama 3 Instruct models that had not been fine-tuned. These preliminary evaluations were performed by the research team.

It was sometimes obvious which model produced a worse output, for example, if one failed to follow the desired format, or copied verbatim from scratch notes. However, in other comparisons, each model had strengths and weaknesses that had to be examined more systematically. Thus, independent evaluations were performed by 3 members of the research team.

For these evaluations, 10 of the curated scratch notes were used, paired with ground-truth SOAP notes, and SOAP notes generated by 2 of the best models. The research team voted on which of the generated outputs was superior overall, considering realism, accuracy, clarity, proper formatting, the extent to which the model followed instructions (eg, “Use point-form”), readability, and qualitative similarity (in terms of voice and style) with the ground-truth SOAP note.

### Pilot Study

#### Overview

The best-performing model, as determined by the qualitative evaluation, was incorporated into a web-based user interface and piloted with a group of 10 occupational therapists at KidsAbility. The goal of the pilot was to compare timing and quality measures between the traditional manual process, the custom model, and Microsoft’s Copilot application, which is based on GPT-4. Due to constraints such as the timing of the study and the availability of clinicians, the pilot was sequential, noncrossover, and nonrandomized. The occupational therapists first wrote all their SOAP notes using Copilot for3 weeks. For each SOAP note, they submitted an online form that indicated the time and date of the note and the self-reported time spent writing it. After a week break from the pilot during which clinicians could either continue using Copilot or revert to a manual process, the clinicians used the custom model for a period of 3 weeks. In this stage, our web application automatically saved their scratch notes, generated draft SOAP notes, and edited SOAP notes. As before, they submitted a form with self-reported time spent on each note. This self-reported time included time typing scratch notes, interacting with the model, and editing the generated SOAP note. All notes required clinician review and editing before being submitted.

#### SOAP Note Quality Evaluation

SOAP note quality was evaluated by a separate research group to reduce bias [62]. Briefly, 4 clinicians, each with at least 2 years of experience in providing pediatric rehabilitation treatment, independently reviewed 256 SOAP notes. The SOAP notes included 64 notes from 4 different categories: manually written SOAP notes (Non-AI Notes), notes written by Copilot and edited by the clinicians (Copilot Edited), unedited SOAP notes from the custom model (Custom), and notes from the custom model that had been edited (Custom Edited). Each clinician was given a rubric with 5 criteria (Clear, Complete, Concise, Relevant, and Organized) on which to score the 256 SOAP notes and a spreadsheet that contained the SOAP notes in a randomized order. The clinicians were not informed of the category of each note, although this may sometimes have been apparent from the note contents, as the manual notes varied more widely in style.

#### Objective Timing Data

Although self-reported timing data were collected during the pilot, the research team sought more precise timing data. Therapists often paused or switched to other tasks while working on SOAP notes, so that automated time stamps would not accurately reflect the time used to write the notes. To collect more accurate timing data, an experimenter observed clinicians and collected timing data in virtual meetings. Four clinicians participated and were asked to write 2-5 SOAP notes per meeting. The clinicians were asked to refrain from beginning their note, including typing any scratch notes, prior to the call. During the virtual meeting, a method for writing each note was chosen at random, with a 50% chance of using the custom model or manually writing the note. A timer was started when the occupational therapist had opened the application they intended to use and were ready to start typing. The timer was stopped once the occupational therapist reported submitting the note in the AlayaCare EMR.

## Results

### Scratch Note Generation

**Figure 2.**
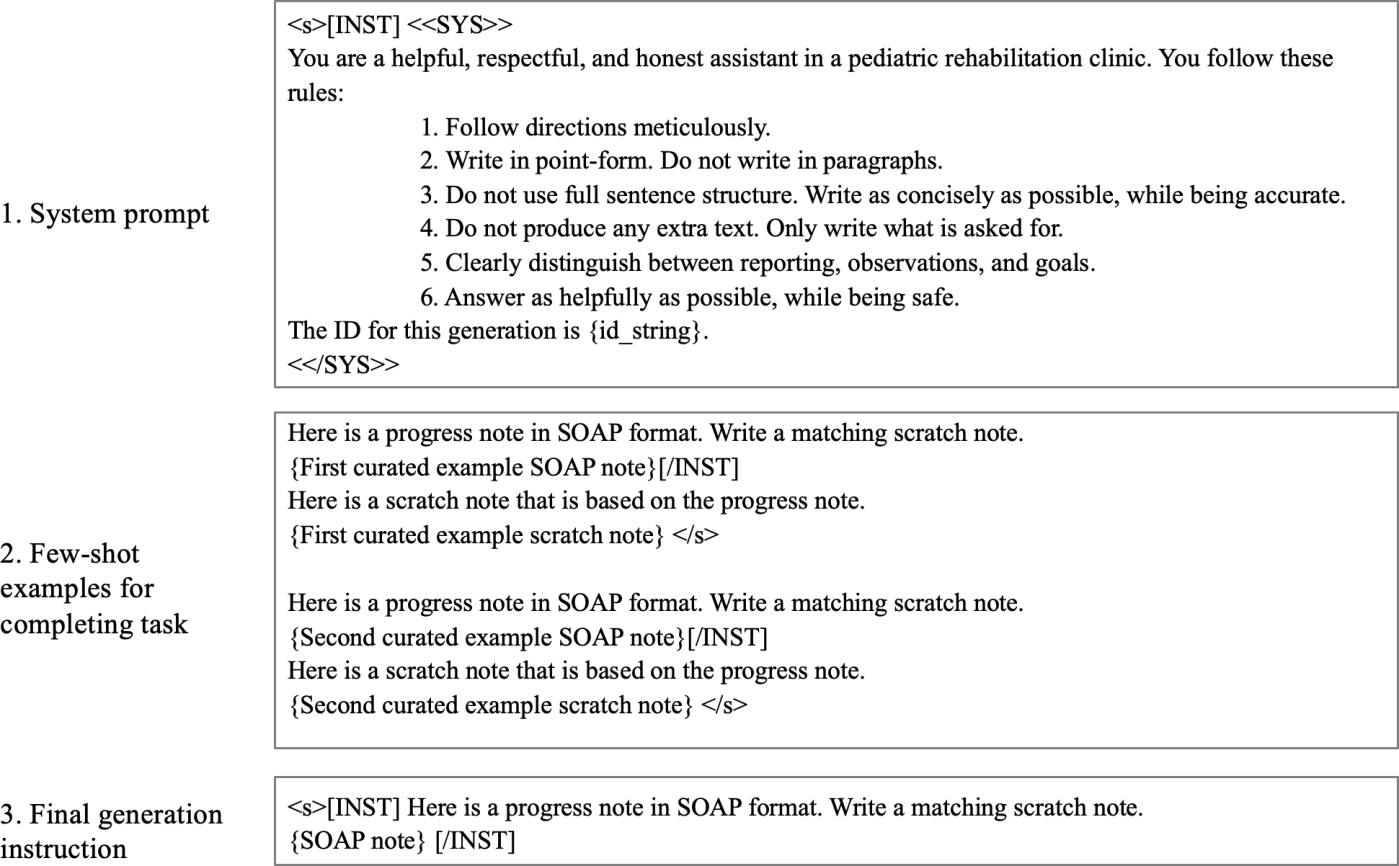
Final prompt structure used for scratch note generation. SOAP: subjective-objective-assessment-plan.

The best variation of few-shot prompting achieved average scores of 4.55 for realism, 4.15 for accuracy, and 4.3 for appropriate distinctions between sections. The worst variation tested achieved scores of 3.5, 3.45, and 3.4 in these categories. The most important factor in performance was the system prompt. The best system prompt is shown in Figure 2. An enumerated list of explicit requirements was effective. Also, when this prompt was used repeatedly on the same input, the outputs received consistently high scores. The instructional prompt and specific few-shot examples had less impact.

### Model Training Metrics

DAPT reduced the training loss metric for the Llama 2 7B Chat model from 2.5 at the beginning of training to 1.4 at the end, suggesting that this training improved the model’s ability to perform CLM on historical progress notes. Table 1 shows the reduction in loss with training for each fine-tuned model as well as the corresponding training times. The impacts of DAPT and LoRA versus full fine-tuning with Llama 2 on the GoldCare dataset were evaluated first. The loss values suggested that DAPT was detrimental to fine-tuning, and that full fine-tuning outperformed LoRA (Table 1). Full fine-tuning without DAPT with both Llama 2 and Llama 3, on both the GoldCare and AlayaCare datasets, was subsequently tested. All the fine-tuned models showed consistent improvement in training and evaluation loss across all training runs.

**Table 1.** Fine-tuning: runtime and impact on loss.

Dataset	Model	Final training loss (as percent initial training loss)	Evaluation loss (as percent initial training loss)	Training runtime, seconds
AlayaCare	Fine-tuned Llama 3	82.5	55.8	715
AlayaCare	Fine-tuned Llama 2	84.6	73.0	639
GoldCare	Fine-tuned Llama 2	73.2	88.8	580
GoldCare	LoRA^a^ and fine-tuned Llama 2	93.5	84.0	586
GoldCare	DAPT^b^ and fine-tuned Llama 2	89.4	83.1	1727
GoldCare	DAPT and LoRA fine-tuned Llama 2	93.5	84.0	586

a LoRA: low-rank adaptation.

b DAPT: domain-adaptive pretraining.

### Qualitative Analysis of Model Outputs

Consistent with the higher loss of the DAPT vs non-DAPT Llama 2 model (Table 1), the outputs from the DAPT Llama 2 model were of low quality, even compared with the original Llama 2 7B Chat model. The DAPT model produced notes that were inappropriately short, missed important details, and often misrepresented the content of the provided scratch note. In comparison, the original Llama 2 model produced notes that were the appropriate length and format, and which better reflected the scratch note’s information, despite also missing important details and misrepresenting information. Final training and evaluation loss are presented as percentage of first-recorded loss because absolute loss values are not comparable between Llama 2 and Llama 3. Lower percentages are better. Fine-tuning was successful in decreasing loss in each case. Fine-tuning was computationally inexpensive.

The fine-tuned Llama 3 model generated outputs that demonstrated the following strengths: an ability to consistently format the notes as desired, with the SOAP note headings and use of bullet points, as well as an ability to write clear, easy-to-read text. However, the Llama 3 model did not always organize the information well into the different sections. For instance, the model sometimes put observations in the subjective section. Meanwhile, SOAP notes generated with the Llama 2 fine-tuned models tended not to use bullet points despite instructions to do so, did not use the correct headings when trained on the GoldCare dataset (where many of the examples did not have SOAP headings), and were less readable than the Llama 3 model’s notes. Notes produced by the Llama 2 models tended to repeat what was written in the given scratch note verbatim more often than the Llama 3 model.

The LoRA fine-tuned Llama 2 model also tended to produce text that was repetitive, not self-consistent, and lacking correct SOAP headings despite explicit instructions. Models that were pretrained on domain data and then fine-tuned with and without the use of LoRA tended to include extra text that was not present or indicated in the scratch note. An example of such irrelevant text that was often included is, “All appropriate COVID-19 precautions taken,” despite the scratch notes indicating nothing to this effect.

When comparing the LoRA fine-tuned models with the equivalent fully fine-tuned models, the outputs of the LoRA models generally included text that was unnecessarily verbose and were more likely to include hallucinations. The fully fine-tuned models tended to be more concise and to stop at the appropriate point in generation.

From these observations, it was clear that the best-performing models were fully fine-tuned Llama 2 and Llama 3. To compare these models more systematically, each model was used to generate SOAP notes from 10 different scratch notes. Three researchers (RD, TT, and TY) reviewed these 10 pairs of notes and voted independently for whether the Llama 2 or Llama 3 note performed best in each case. Fine-tuned Llama 3 notes received 20 votes in total, whereas fine-tuned Llama 2 received 10, suggesting that Llama 3 was stronger overall. Sample-generated notes can be seen in Multimedia Appendix 2. Thus, the fine-tuned Llama 3 8B Instruct model (hereafter referred to as the custom model) was chosen for the pilot.

### Pilot Study Findings

#### Qualitative Feedback

At the start of the KidsAbility Pilot Study, much of the feedback about the custom model included actionable suggestions for improvement. Clinicians requested that the model use a more consistent bullet point marker, which was quickly addressed by adding a find-and-replace function to the code. They also noted that the model produced high-quality plan and analysis sections, especially compared with the Copilot model, and that the text it generated was professional, concise, readable, and familiar in tone. Sample-generated notes can be seen in Multimedia Appendix 2 listings 1 and 3 for Llama 3 and Copilot, respectively. Negative feedback included frustrations with the editing process, incorrect placement of information under headings, occasional omissions of details from scratch notes, and rare instances of irrelevant or incorrect text being added.

Subjective reporting of the time taken to write SOAP notes with the custom model varied from 5 to 26 minutes, with an average of 13.83 (SD 7.10) minutes (n=73). In comparison, the average time for using Copilot was 14.08 (SD 8.99) minutes (n=190). Significant variability in the time required to complete notes with both models was reported, and the difference was not statistically significant between models.

#### Dependence of Time Savings on Scratch Note Style

Some therapists appeared to use the custom model more efficiently than others. Upon investigation, the KidsAbility Innovation Team found that the 2 therapists who reported the best time savings were providing much shorter scratch notes, while other therapists were spending more time to write out detailed scratch notes for the model that already closely resembled full SOAP notes. Following this discovery, the innovation team held one-on-one sessions with 6 of the clinicians, coaching the clinicians to provide sparser scratch notes to the model and to spend less time writing these. Examples of a detailed scratch note and a sparse scratch note can be seen in Multimedia Appendix 3.

The average time taken after this coaching, based on self-reporting, was 7.6 (SD 3) minutes (n=6). An example from one of the coaching sessions illustrates the potential impact of coaching. Initially, a clinician spent 10 minutes writing detailed scratch notes, including SOAP headings. After one-on-one coaching, the clinician produced a much sparser version of the scratch notes in 1 minute, and the entire process was completed in approximately 6.5 minutes. While anecdotal, these observations suggest that the model has the potential to reduce documentation time, but the actual time savings depend strongly on how the model is used.

#### Comparison of Unedited and Edited Model Notes

The clinicians at KidsAbility were accountable for reviewing all generated notes and editing them as appropriate. ROUGE (Recall-Oriented Understudy for Gisting Evaluation) scores [63] were calculated to compare each edited model note against the unedited, generated note.

ROUGE scores measure similarity between a piece of text and a reference piece of text. ROUGE-1 scores are *F*_1_-scores for which the recall and precision are calculated using the number of overlapping words. While ROUGE-1 scores provide a measure of the similarity based on the number of individual words that are the same, ROUGE-2 scores are based on 2-word sequences. ROUGE-L and ROUGE-LSUM scores indicate sentence-level similarity and similarity at the level of the entire note, respectively [63,64]. A higher score indicates a higher degree of similarity and, in this case, less editing.

Sixty-four pairs of edited and unedited notes were compared. The means and SDs of 4 different ROUGE metrics calculated for all model output notes compared with the final edited notes are shown in Table 2. All the ROUGE scores were high, indicating light editing at both the word and document levels. Somewhat lower ROUGE-2 and ROUGE-L scores may indicate that most changes are related to word order and sentence structure.

**Table 2.** ROUGE^a^ scores comparing custom model notes before and after editing by clinicians (higher scores mean less editing at various levels of granularity).

	Score, mean (SD)	95% CI
ROUGE-1	0.71 (0.17)	0.67-0.75
ROUGE-2	0.58 (0.25)	0.52-0.64
ROUGE-L	0.63 (0.24)	0.57-0.69
ROUGE-LSUM	0.70 (0.18)	0.65-0.74

a ROUGE: Recall-Oriented Understudy for Gisting Evaluation.

#### SOAP Note Quality Assessment

The mean scores for the 5 criteria across the 4 different groups of SOAP notes are shown in Table 3, and the corresponding SDs are shown in Table 4. The Custom Edited notes had the highest or second-highest scores for all 5 criteria. Meanwhile, the Non-AI notes were consistently scored lowest except on the Conciseness criterion. Finally, the custom model notes had the lowest SDs across all criteria, with the Custom Edited notes having the lowest for 4 out of 5. It is possible that the low SD indicates higher consistency in quality.

**Table 3.** Mean scores (out of a maximum of 3) assigned to SOAP^a^ notes within each note type category.

Note type	Clear	Complete	Concise	Relevant	Organized
Non-AI^b^ note	2.24	1.94	2.57	2.61	2.06
Copilot Edited	2.65	*2.66* ^c^	2.34	2.77	*2.51* ^c^
Custom	2.50	2.55	2.55	2.76	2.33
Custom Edited	*2.66* ^c^	2.61	*2.57* ^c^	*2.80* ^c^	2.49

a SOAP: subjective-objective-assessment-plan.

b AI: artificial intelligence.

c The highest mean scores for each criterion are in italics.

**Table 4.** SD of the scores assigned to SOAP^a^ notes averaged across the different scorers within each note type category.

Note type	Clear	Complete	Concise	Relevant	Organized
Non-AI^b^ note	0.59	0.62	0.57	0.52	0.60
Copilot Edited	0.54	0.54	0.65	0.40	0.59
Custom	0.54	0.52	*0.56* ^c^	0.43	0.64
Custom Edited	*0.48* ^c^	*0.46* ^c^	0.57	*0.33* ^c^	*0.56* ^c^

a SOAP: subjective-objective-assessment-plan.

b AI: artificial intelligence.

c The lowest SD for each criterion is in italics.

Two-factor ANOVA tests with replication on each of the 5 criteria were performed, with the 2 factors being the clinician evaluator and the category of note. Both main factors were significant with *P*<.001 across all criteria (17.37 ≤ *F*_3,1004_ ≤126.17). There were also moderate interaction effects between the 2 factors for all criteria, the greatest of which was *F*_9,1004_=12.16, *P*<.001 for clarity. The ANOVA test was appropriate as a robust omnibus test for Likert-type ratings given the large, balanced sample and approximately symmetrical residuals.

Pair-wise comparisons between scores of different note categories were performed using Tukey test to correct the significance threshold for multiple comparisons. These tests showed that there was a significant difference (0.25‐0.77, *P*<.001) in mean scores between the Non-AI notes and the Custom Edited notes for all criteria except for conciseness. Conversely, there was a significant difference (0.31, *P*<.001) between the mean scores for the custom model notes and Copilot notes only for conciseness. See Multimedia Appendix 4 for further details.

Interrater reliability was measured using the intraclass correlation coefficient (ICC) metric. The metric varied by criterion, with single-rater reliabilities being low (ICC_3,1_=0.16-0.41), indicating limited interchangeability of clinician evaluators [65]. In contrast, reliability of the average rating across clinicians was higher (ICC_3,4_=0.43-0.74).

#### Objective Timing Data

Online meetings were used to record more accurate timing data for a small number of notes. A member of the research team timed the note-taking duration of 18 instances, including 10 using the custom model and 8 manually written. The average time spent on writing notes with the custom model was 9.55 (SD 3.5) minutes (n=10). The manually written notes took an average of 7.84 (SD 3.1) minutes (n=8). Of note is the large degree of variability in the times recorded for both categories of notes. Varied complexity of different notes was a factor in this variation. For example, for one of the custom model notes, the clinician commented that it was much more complex than usual and could be considered an outlier. Similarly, the clinician who manually wrote another note commented that it was shorter than usual because it was for a brief virtual call that did not contain any interaction with the client. Excluding these 2 outliers, the average time taken to write notes with the custom model was 8.76 (SD 2.6) minutes, while the average time for the manual process was 8.38 (SD 2.9) minutes. This difference was not statistically significant.

Despite the fact that 3 of the 4 clinicians who participated had previously gone through one-on-one coaching to use the model more effectively, they did not appear to implement the more effective strategy. The scratch notes were longer and more detailed than they had been coached previously to write.

## Discussion

### Overview

Non–direct administrative tasks are a substantial burden in pediatric rehabilitation, contributing to burnout [12], as well as reducing therapist capacity and contributing to growing waitlists for care. A quick fix for this issue is not apparent. Therapists perform a variety of administrative tasks that may require varied interventions. Documentation cannot be completely automated because therapists remain accountable for the accuracy and completeness of the documentation, because existing technology produces errors, and because some of the relevant information may be known only to therapists. Producing progress notes is a substantial administrative task, taking about one-third the time of direct care. The recent success of automated documentation in other clinical settings suggests that LLMs may be able to improve the efficiency of this process, an important step in reducing administrative burden overall. A customized LLM was developed and tested for this application, motivated by the goals of optimizing performance, keeping PHI under the control of the health care institution, and minimizing the climate impact associated with use of the model.

### Governance and Ethical Considerations

The development and deployment of the custom model were overseen by the KidsAbility Innovation team, and model access was restricted to clinicians within a secure, institutionally authorized environment. System logging was implemented, with data stored securely, enabling review and auditing of generated notes. The clinicians remained legally responsible for the content of the final SOAP notes, with the model notes considered drafts. They were required to review and edit every draft progress note. While the custom model was the best-performing model of those tested during this study, it still produced irrelevant boilerplate text or hallucinations on occasion, so it was imperative that the clinicians carefully reviewed the notes for such errors and removed them in their editing process.

Several ethical risks were considered in development, including data privacy risks, which were mitigated by deidentifying the training data and removing PHI from note text. Another risk for such a system could be automation bias or clinician overreliance, which was addressed largely by emphasizing the need for human review and accountability. The draft status of the generated notes was made clear, and the clinician was responsible for finalizing or submitting any data to the EMR. Finally, fairness considerations meant that the model was not assumed to generalize across settings, given that the data reflect only KidsAbility patient demographics and documentation practices. Moving forward, it will be important to monitor the model’s outputs to ensure that there are no systematic errors or biases.

### Principal Findings

#### SOAP Note Quality Measures

A surprising result was that progress notes that were produced using this model scored better on multiple quality measures than manually written notes. Specifically, compared with SOAP notes written without the use of LLMs, those written with either Copilot or the custom model were given higher scores for all 5 criteria, apart from Conciseness. Non-AI notes received higher scores for Conciseness than the Copilot model notes but not the edited custom model notes. Therefore, the use of LLMs did not degrade the quality of the SOAP notes but instead helped to produce higher quality SOAP notes.

Progress notes produced with the custom model also compared favorably with progress notes produced with Microsoft Copilot. Comparing the notes generated with the custom model with the notes generated with the Copilot model, the custom model received higher scores for conciseness regardless of editing, but otherwise the scores for edited notes of both models were similar. Clinicians informally corroborated this finding, reporting that the Copilot notes were longer and contained more “fluff” than those from the custom model.

#### Time Savings

The impact of the models on therapists’ time was less clear. During the pilot study, it was found that self-reported time savings varied substantially between therapists. Further investigation suggested that this variation related to different ways in which different therapists used the model. Most clinicians in the pilot wrote long, detailed scratch notes and still spent significant time editing the generated notes. This approach was inefficient, as the model could only rephrase or rearrange points without adding meaningful content. Therapists who used the model more effectively found that writing quick, minimal scratch notes allowed the model to produce a usable draft, which they could then edit. After being coached by the research team, all therapists reported better results and were able to create quality SOAP notes in less time, demonstrating the importance of hands-on training to maximize the model’s effectiveness.

It is unsurprising that writing more detailed scratch notes would reduce the benefit of the system but perhaps more surprising that many therapists were inclined to do so. During the objective timing study, several therapists were observed to have reverted to giving the model long, detailed scratch notes despite previous coaching and positive experience providing short, sparse notes. Further work is needed to understand how best to encourage more efficient use of the system. For example, this could involve user interface changes to show an ideal example, or a limit on the number of words that can be entered in the scratch note interface. Clinicians specifically expressed a wish to provide further prompts or instructions to the model for a given note, which could improve model usability with sparse scratch notes. Alternatively, an analysis of model limitations that may be driving provision of longer scratch notes could be pursued. It is possible that the synthetic training data, which were more well-structured and clearer than typical manual scratch notes, may contribute to model limitations by introducing a bias toward longer and more complete scratch notes. Further fine-tuning of the model on the clinicians’ scratch notes and the progress notes produced by the model and edited by clinicians could improve model performance. If the model with further fine-tuning were to produce progress notes more aligned with their preferences given sparse scratch notes, clinicians may be less inclined to provide overly long scratch notes. Overall, work to promote efficient use of the system appears to be critical for realizing the efficiency gains that would be needed to impact waitlists for treatment. However, even if the model were used optimally, impact on waitlists would require therapists to take on larger caseloads.

Clinicians’ previous experiences with the Copilot model may also have contributed to their initial, less effective use of the custom model. Some had developed biases, believing that detailed input was necessary for generating useful SOAP notes, as they were dissatisfied with Copilot’s Analysis and Plan sections when sparse notes were provided. As a result, some clinicians used only Copilot for the Subjective and Objective sections, preferring to write the other parts themselves. This may have influenced their approach to the new model.

### Model Development Lessons

#### DAPT

The models that underwent DAPT experienced catastrophic forgetting, where their ability to respond to prompts worsened after continued training. See Multimedia Appendix 5 for an example of degraded performance. Specifically, DAPT caused the models to ignore instructions, similar to findings by Cheng et al [66], who describe a similar phenomenon in their paper, proposing the use of reading comprehension tasks to reduce this issue by training the model on more diverse prompts. However, their methodology was impractical for reframing the SOAP note dataset. Thus, it is hypothesized that a lack of diversity in the DAPT prompts contributed to the degradation in performance, as the training examples did not contain any explicit instructions and merely presented SOAP notes.

Huang et al [67] also propose a mitigation strategy for catastrophic forgetting due to continued training, using self-synthesized data that are meant to reflect the original training data to retain the model’s original proficiencies. Future work on this project could use similar methods to include other types of examples, reflective of original training examples, in the process of DAPT along with the examples of SOAP notes.

Öncel et al [68] explored factors that play a role in whether performance degrades with further training. They found that greater similarity between the original training corpus and the new domain’s corpus resulted in more degradation. Given the breadth of the Llama training corpus for Llama 2, it is possible that the original data the model was trained on contained examples very similar to our DAPT data. Öncel et al also found that increasing the model size could mitigate performance degradation. Therefore, it is hypothesized that the small size of the model (relative to other language models) played a role in the observed catastrophic forgetting. An interesting avenue for future work could include training the model on smaller, more curated datasets and determining when catastrophic forgetting begins to emerge.

Additionally, the models that underwent DAPT were more prone to hallucinating irrelevant text in the SOAP notes, perhaps due to low variety in the DAPT examples. The model often generated repetitive phrases such as COVID-19 protocol text instead of responding dynamically to the scratch notes. Öncel et al [68] make a relevant observation in their study, observing that the models tend to improve at predicting domain-specific tokens and to become worse at generic tokens, especially structural tokens. The COVID-19 protocol appears frequently in training data and so may be seen by the model as a set of domain-specific tokens that should be predicted often.

#### Parameter-Efficient Fine-Tuning

LoRA fine-tuning was faster than full fine-tuning (9.7 minutes vs 28.9 minutes on the GoldCare fine-tuning dataset). Merging adapter weights back into the model added 12 minutes on average. However, despite a minor speed advantage, LoRA fine-tuned models had higher training and evaluation losses than fully fine-tuned models.

Consistent with these higher losses, the LoRA-trained models generated SOAP notes that were less concise, often overly long, and prone to hallucinating irrelevant or inappropriate text. Many notes failed to follow the correct SOAP format and sometimes included repetitive or nonsensical sections, with placeholder tokens such as “[GOAL]” or “[DATE]” appearing in the note. These issues made the LoRA fine-tuned models less suitable for this task than fully fine-tuned models.

#### Llama 2 Versus Llama 3

Comparing the generated notes of the Llama 2 and Llama 3 fine-tuned models, the Llama 3 model generally produced more readable notes. The Llama 3 model was more likely to change the wording of a particular point in a scratch note to improve clarity, while still conveying the original meaning. Furthermore, a large difference between the 2 models was that the fine-tuned Llama 3 model responded more appropriately to instructions after training. The fine-tuned Llama 2 model, like the DAPT models, demonstrated reduced ability to respond to instructions [66], most likely due to training on a narrower task. However, the Llama 3 model did not exhibit decreased performance in responding to instructions, instead responding appropriately to different prompts even after fine-tuning. In this way, the fine-tuned Llama 3 model could be prompted to, for instance, use specific headers only or be of a more specific length.

### Limitations

#### Workflow Limitations

Clinician availability was a limiting factor in this work due to their existing workload. Clinicians were not available for extensive evaluation of the model outputs, nor was there time for clinicians to produce scratch notes for use in training the model. Furthermore, the sequential, nonrandomized nature of the pilot study potentially introduced order effects such that the use of the Copilot model prior to use of the custom model may have influenced how the clinicians used the custom model. Comparative performance and time-saving metrics between the 2 models should be interpreted with this in mind.

The impact of the system on therapists’ time was unclear. Timing data from the pilot study were self-reported. The team made a preliminary attempt to collect more accurate timing data through online meetings. A statistical power analysis with G*Power indicated that a sample size of 176 for each category (total 352 data points) would be needed to find a moderate difference. Thus, a much larger sample would be required, and the described findings cannot be considered definitive. However, the limited data collected, and the fact that therapists had reverted to an inefficient process, argue against substantial time savings. In future work, it would be best to revise the process so that therapists provide sparse scratch notes before collecting a larger timing dataset. Additionally, future work could include clinician satisfaction ratings or other usability metrics and feedback to report more thoroughly on the clinician experience outside of time taken to write notes.

#### Model Limitations

Regarding model performance, the choice to train for only a single epoch may have resulted in poorer performance than could have been achieved with further training (eg, with 3 epochs as commonly suggested for such fine-tuning) [69,70]. There exists an inherent trade-off that in limiting the number of epochs to mitigate memorization and privacy risks, the model’s performance and generalizability will likely be poorer. Also, the use of synthetic scratch notes may have resulted in bias and degraded performance as well.

Finally, this study was focused only on OT SOAP notes for a single institution, and it is not clear whether the same type of model would be generalizable to other settings or disciplines such as physiotherapy. The model would likely not be as useful for other organizations, given that it is trained to mimic the style and culture of the therapists at KidsAbility. A model for a different program at a different organization or for a new discipline would require adaptations but could likely be developed following the same general procedure.

### Conclusions

Our customized LLM, which was fine-tuned on pairs of historical progress notes and corresponding synthetic scratch notes, produced progress notes that were similar or better than manually written notes in all the evaluated quality dimensions. Copilot performed similarly but was less concise. Copilot’s technical details are not public, but it is likely more resource-intensive to run, by perhaps 2 orders of magnitude. Fine-tuned models such as this have the potential to help health care practitioners in specialized domains to write high-quality documentation, in a cost-effective and efficient manner, without sending PHI to third parties.

It appeared from the analysis of pilot data and coaching after the pilot study that the model could reduce time spent writing progress notes but only if therapists provided short, sparse scratch notes and spent most of the time editing the generated note. Coaching several therapists to use the model in this way appeared to help them produce progress notes faster. However, the therapists reverted to writing detailed notes during the collection of timing data, and no statistically significant time reduction was found when using the custom model compared with the manual process. When writing longer, more detailed scratch notes, the model primarily improved quality rather than efficiency. Altogether, time savings seemed to depend strongly on therapists letting the model elaborate sparse scratch notes, but they often seemed disinclined to do so.

To further improve generated progress notes, the model could undergo further fine-tuning on pairs of scratch notes and edited progress notes on an ongoing basis. This might result in progress notes that are even more accurate and more in line with clinician preferences.

## Supplementary material

10.2196/73274 Multimedia Appendix 1 Model training implementation and hyperparameters.

10.2196/73274 Multimedia Appendix 2 Generated subjective-objective-assessment-plan note examples.

10.2196/73274 Multimedia Appendix 3 Scratch note examples.

10.2196/73274 Multimedia Appendix 4 ANOVA and intraclass correlation coefficient results for subjective-objective-assessment-plan note quality assessment.

10.2196/73274 Multimedia Appendix 5 Domain-adaptive fine-tuning example.

## Abbreviations

AI: artificial intelligence
CLM: causal language modeling
DAPT: domain-adaptive pretraining
EMR: electronic medical record
ICC: intraclass correlation coefficient
LLM: large language model
LoRA: low-rank adaptation
OT: occupational therapy
PHI: personal health information
ROUGE: Recall-Oriented Understudy for Gisting Evaluation
SOAP: subjective-objective-assessment-plan
