# Supplementary material for: A Language Model for Pediatric Occupational Therapy Documentation: Model Development and Pilot Study
Source: JMIR AI. 2026 May 15;5:e73274. doi: 10.2196/73274 (PMC13179052; doi:10.2196/73274)
Supplement: Multimedia Appendix 1 [file ai-v5-e73274-s001.docx]

**Multimedia Appendix 1. Model training implementation and hyperparameters.**

**Model training implementation**

All model training was performed using an AWS SageMaker notebook with a DeepSpeed Launcher for training on a SageMaker Training Job Instance. The instance had 8 V100 GPUs. HuggingFace’s Supervised Fine-Tuning Trainer handled the training loop. Below are the hyperparameters used for the two types of training performed.

For DAPT, a system prompt was appended to all training examples, specifying that the given note was a SOAP note. The appended prompt for fine-tuning was longer, and each example contained a scratch note and a progress note, which was why a longer sequence length was used for fine-tuning.

**Training Hyperparameters**

| Hyperparameter | DAPT | Fine-Tuning |
| --- | --- | --- |
|  |  |  |
| Learning Rate | 5 x 10^-5^ | 5 x 10^-5^ (0.05 warmup ratio) |
| Per-device Batch Size | 2 | 2 |
| Gradient Accumulation Steps | 2 | 2 |
| Sequence Length | 1024 tokens | 4096 tokens |
| Epochs | 1 | 1 |
