# Supplementary material for: A Language Model for Pediatric Occupational Therapy Documentation: Model Development and Pilot Study
Source: JMIR AI. 2026 May 15;5:e73274. doi: 10.2196/73274 (PMC13179052; doi:10.2196/73274)
Supplement: Multimedia Appendix 2 [file ai-v5-e73274-s002.doc]

## Multimedia Appendix 2. Generated SOAP note examples.

Following are examples of SOAP notes generated by various systems. Text in square brackets is a description of text that is redacted to maintain privacy.

### Fine-tuned Llama 3 model

Subjective

* SERT concerns about chair size

* Chair too small for him

* Tried chair with desk, desk too tall

* Tried with shorter desk, seemed better

Objective

* Met with OT to discuss options

* Tried chair with [Size] desk, seemed to work OK

* School reports he still likes using chair

* Marked desk and requested custodian to lower it or swap to lowered size

* Rocker chair still appropriate

* Larger size available within school

Analysis

* Noted that chair size may be a concern for SERT but still seems to be working OK for [NAME] with [SIZE] desk

* SERT will follow up as needed

Plan

* Monitor chair use and adjust as needed

* Follow up with SERT as needed

### Llama 2 model fine-tuned with AlayaCare dataset

Subjective

[NAME] report that [NAME] is still using rocker chair, but if chair is too small [NAME] will use the bigger one that is used by [OTHER STUDENT]

Objective

OT met with ct and tried the chair. Desk was much too tall. Tested with shorter desk and it seemed to be better.

[NAME] reports that [Size] is still OK and is still smaller than peers but it works out. Ct reports that he still likes the chair.

Analysis

[NAME] reported that desk should be lowered or swapped to a lower size to accommodate chair.

Chair is appropriate size for student.

No f/u required at this time.

Plan

OT to follow up as needed.

### Copilot

Subjective:

School visit [Date]. Occupational Therapist (OT) met with the teacher and observed the student in class. The teacher reports that the when provided with teacher’s attention meltdowns get prolonged and the crying gets louder. The student is able to verbalize the reason for the upset after the incident.

Objective:

[NAME] approached the teacher for help. [NAME] was able to formulate a complex sentence when scribed. Good printing legibility was observed.

Analysis:

The teacher suggests that [NAME] should not take the [TEST]. [NAME] struggles significantly ([BEHAVIOURS]) with any kind of testing (e.g., short spelling tests). [TEST] may be too much for [NAME].

Sensory tools are currently in use. A stand-up desk is also being utilized.

Plan:

The OT will continue to monitor [NAME]'s progress and make necessary adjustments to the plan as needed.
