# Supplementary material for: A Language Model for Pediatric Occupational Therapy Documentation: Model Development and Pilot Study
Source: JMIR AI. 2026 May 15;5:e73274. doi: 10.2196/73274 (PMC13179052; doi:10.2196/73274)
Supplement: Multimedia Appendix 3 [file ai-v5-e73274-s003.docx]

**Multimedia Appendix 3.** Scratch note examples.

Following are examples of scratch notes that illustrate the difference between long, detailed scratch notes and sparse, short scratch notes. Also included are the unedited and edited SOAP note generated by the custom model using the sparse scratch note example.

**Long, detailed scratch note example**

S:

-not interested in food in pillow case

-likes vibrating pillow, brings to face

-has mom cover his ears w/ loud noises

-visual stimming on danglers

-flight risk at park

-holding oranges all day until +++soggy

-continues to feed [SIBLING] purees

-sticks fingers in ketchup

-lining up shoes/toys at home

-[LOCATION] F/U in [MONTH]

O:

-enjoyed chase, deep pressure squeezes and puppet eating his arm

-requesting more by offering limbs w/ deep pressure or smiling w/ chase

-allowing OT to squeeze his face, not liking OT tickling his face

-played w/ blocks, stacking and taking turns w/ OT.

-visually stimming on dangling elastic/tubing throughout session

-recommended Mom work on squeezes/tickling back of head/ears/working towards face.

A:

-tolerating deep pressure around his face, showing more engagement w/ deep pressure. Continues to tolerate touch of different foods

P:

-see next week w/ SLP

-continue w/ goal of desensitizing around face/mouth

-mom in agreement w/ plan

**Short, sparse scratch note example**

- Likes vibrating pillow, brings to face

- Visual stimming on danglers

- Flight risk at park

- Enjoyed chase, deep pressure squeezes, and puppet play

- Requested more by offering squeez on limbs or smiling chase

- Played with blocks, stacking, and taking turns with OT

- Tolerats + liked deep pressure around his face

- still tolerated touching diff food

- See next week with SLP

- Continue goal of desensitizing around face/mouth

- Mom agrees with plan

**SOAP note generated by the custom model using the short, sparse scratch note example**

Subjective:

- Likes vibrating pillow, brings to face

- Visual stimming on danglers

- Requested more by offering squeezes on limbs or smiling chase

- Enjoyed deep pressure squeezes and puppet play

Objective:

- Played with blocks, stacking, and taking turns with OT

- Tolerated and liked deep pressure around his face

- Still tolerated touching different foods

Analysis:

- Visual stimming on danglers continues to be a source of comfort

- Request for more stimulation by offering squeezes on limbs or smiling chase indicates that deep pressure is still a source of relaxation

- Continues to tolerate and enjoy deep pressure around face

- Goal of desensitizing around face/mouth remains relevant

Plan:

- Continue offering deep pressure squeezes and smiling chase

- Continue playing with blocks, stacking, and taking turns with OT

- Continue working on tolerating touching different foods

- See next week with SLP

- Continue to monitor and adjust plan as needed

**Edited SOAP note generated by the custom model using the short, sparse scratch note example (clinician edits shown in italics)**

Subjective:

- Likes vibrating pillow, brings to face

- Visual stimming on danglers

- Requested more by offering squeezes on limbs or smiling chase

- Enjoyed deep pressure squeezes and puppet play

*- Stuck fingers in ketchup*

*- Flight risk at the park*

*- holding oranges all day until soggy ++*

*- continues to feed brother puree*

*- lining up shoes/toys in order at home*

Objective:

- Played with blocks, stacking, and taking turns with OT

- Tolerated and liked deep pressure around his face but not liked OT tickling face.

- Still tolerated touching different foods

*- enjoyed chase and deep pressure squeezes with puppet eating his arm*

*- requesting more by offering deep limb pressure and smiling with chase*

*- visual stimming on dangling elastic throughout session*

Analysis:

- Visual stimming on danglers continues to be a source of comfort

- Request for more stimulation by offering squeezes on limbs or smiling chase indicates that deep pressure is still a source of relaxation

- Continues to tolerate and enjoy deep pressure around face

Plan:

- Continue offering deep pressure squeezes and smiling chase

- Continue playing with blocks, stacking, and taking turns with OT

- Continue working on tolerating touching different foods

- See next week with SLP

- Continue to monitor and adjust plan as needed

*- [LOCATION] F/U in [MONTH]*

*- Recommend mom to continue deep pressure squeezes, and tickling working from back of head towards face*

- Goal of desensitizing around face/mouth remains relevant
