# Supplementary material for: A Language Model for Pediatric Occupational Therapy Documentation: Model Development and Pilot Study
Source: JMIR AI. 2026 May 15;5:e73274. doi: 10.2196/73274 (PMC13179052; doi:10.2196/73274)
Supplement: Multimedia Appendix 4 [file ai-v5-e73274-s004.docx]

**Multimedia Appendix 4.** ANOVA and ICC results for SOAP note quality assessment.

**ANOVA Test**

A two-way ANOVA test with replication was performed for the SOAP Note Quality scores on each of the five criteria. The tables below show the degrees of freedom, test statistic, and p-values for the two factors as well as the interactions between the factors.

***Clinicians***

Table S1. Two-way ANOVA results for clinician evaluators.

|  | df_1_ | df_2_ | F | P-value |
| --- | --- | --- | --- | --- |
|  |  |  |  |  |
| Clear | 3 | 1004 | 120.96 | <.001 |
| Complete | 3 | 1004 | 126.17 | <.001 |
| Concise | 3 | 1004 | 35.23 | <.001 |
| Relevant | 3 | 1004 | 104.39 | <.001 |
| Organized | 3 | 1004 | 43.17 | <.001 |

***Note-Type***

Table S2. Two-way ANOVA results for the note-type.

|  | df_1_ | df_2_ | F | P-value |
| --- | --- | --- | --- | --- |
|  |  |  |  |  |
| Clear | 3 | 1004 | 33.80 | <.001 |
| Complete | 3 | 1004 | 125.87 | <.001 |
| Concise | 3 | 1004 | 19.61 | <.001 |
| Relevant | 3 | 1004 | 17.37 | <.001 |
| Organized | 3 | 1004 | 31.71 | <.001 |

***Interaction Between Clinician and Note-Type***

Table S3. Two-way ANOVA results for the interaction between the two factors.

|  | df_1_ | df_2_ | F | P-value |
| --- | --- | --- | --- | --- |
|  |  |  |  |  |
| Clear | 9 | 1004 | 12.16 | <.001 |
| Complete | 9 | 1004 | 3.25 | <.001 |
| Concise | 9 | 1004 | 10.57 | <.001 |
| Relevant | 9 | 1004 | 6.63 | <.001 |
| Organized | 9 | 1004 | 7.19 | <.001 |

**Tukey Tests**

A pairwise comparison with Tukey’s test was performed for the different note-types. Below are tables that show the mean difference between different note categories, the confidence intervals, and the adjusted p-values, with significant differences in bold font.

Table S4. Legend for the note-types.

| Letter | Note-Type |
| --- | --- |
|  |  |
| K | Non-AI note |
| E | Copilot edited |
| U | Custom |
| T | Custom edited |

***Clear***

Table S5. Pairwise comparison of note-types with Tukey’s test results for clarity criterion.

|  | Mean Difference | 95% CI | Adjusted P-value |
| --- | --- | --- | --- |
|  |  |  |  |
| **K-E** | **-0.36** | **[-0.48, -0.24]** | **<.001** |
| T-E | 0.064 | [-0.053, 0.18] | .49 |
| U-E | -0.096 | [-.0.21, 0.021] | .148 |
| **T-K** | **0.42** | **[0.31, 0.54]** | **<.001** |
| **U-K** | **0.26** | **[0.15, 0.38]** | **<.001** |
| **U-T** | **-0.16** | **[-0.28, -0.044]** | **.0024** |

***Complete***

Table S6. Pairwise comparison of note-types with Tukey’s test results for completeness criterion.

|  | Mean Difference | 95% CI | Adjusted P-value |
| --- | --- | --- | --- |
|  |  |  |  |
| **K-E** | **-0.80** | **[-0.92, -0.68]** | **<.001** |
| T-E | -0.031 | [-0.15, 0.092] | .92 |
| U-E | -0.090 | [-.0.21, 0.034] | .24 |
| **T-K** | **0.77** | **[0.65, 0.89]** | **<.001** |
| **U-K** | **0.71** | **[0.59, 0.83]** | **<.001** |
| U-T | -0.059 | [-0.18, 0.065] | .61 |

***Concise***

Table S7. Pairwise comparison of note-types with Tukey’s test results for conciseness criterion.

|  | Mean Difference | 95% CI | Adjusted P-value |
| --- | --- | --- | --- |
|  |  |  |  |
| **K-E** | **0.40** | **[0.26, 0.55]** | **<.001** |
| **T-E** | **0.31** | **[0.17, 0.46]** | **<.001** |
| **U-E** | **0.30** | **[0.15, 0.44]** | **<.001** |
| T-K | -0.090 | [-0.23, 0.055] | .38 |
| U-K | -0.11 | [-0.25, 0.039] | .24 |
| U-T | -0.016 | [-0.16, 0.13] | .99 |

***Relevant***

Table S8. Pairwise comparison of note-types with Tukey’s test results for relevance criterion.

|  | Mean Difference | 95% CI | Adjusted P-value |
| --- | --- | --- | --- |
|  |  |  |  |
| **K-E** | **-0.21** | **[-0.31, -0.12]** | **<.001** |
| T-E | 0.036 | [-0.062, 0.13] | .77 |
| U-E | -0.015 | [-.0.11, 0.083] | .98 |
| **T-K** | **0.25** | **[0.15, 0.35]** | **<.001** |
| **U-K** | **0.20** | **[0.10, 0.30]** | **<.001** |
| U-T | -0.051 | [-0.15, 0.047] | .54 |

***Organized***

Table S9. Pairwise comparison of note-types with Tukey’s test results for organization criterion.

|  | Mean Difference | 95% CI | Adjusted P-value |
| --- | --- | --- | --- |
|  |  |  |  |
| **K-E** | **-0.42** | **[-0.55, -0.29]** | **<.001** |
| T-E | 0.035 | [-0.099, 0.17] | .91 |
| U-E | -0.085 | [-.0.22, 0.048] | .35 |
| **T-K** | **0.45** | **[0.32, 0.59]** | **<.001** |
| **U-K** | **0.33** | **[0.20, 0.47]** | **<.001** |
| U-T | -0.12 | [-0.25, 0.013] | .095 |

**ICC Test**

An intraclass correlation coefficient (ICC) was calculated for each of the five criteria across the independent raters to measure inter-rater reliability. Both the two-way mixed, single measure, absolute agreement (ICC(3,1)), and two-way mixed, average measures, absolute agreement (ICC(3, 4)) ICC scores were calculated. The following tables show the ICC scores including the 95% confidence intervals and p-values.

Table S10. ICC Values for the five criteria.

|  | ICC Type | Value | 95% CI | P-value |
| --- | --- | --- | --- | --- |
|  |  |  |  |  |
| Clear | ICC(3, 1) | 0.17 | [0.081, 0.263] | <.001 |
|  | ICC(3, 4) | 0.45 | [0.162, 0.625] | .0024 |
| Complete | ICC(3, 1) | 0.40 | [0.207, 0.552] | <.001 |
|  | ICC(3, 4) | 0.73 | [0.464, 0.841] | <.001 |
| Concise | ICC(3, 1) | 0.41 | [0.316, 0.503] | <.001 |
|  | ICC(3, 4) | 0.74 | [0.634, 0.808] | <.001 |
| Relevant | ICC(3, 1) | 0.16 | [0.077, 0.245] | <.001 |
|  | ICC(3, 4) | 0.43 | [0.155, 0.604] | .0024 |
| Organized | ICC(3, 1) | 0.27 | [0.186, 0.356] | <.001 |
|  | ICC(3, 4) | 0.60 | [0.449, 0.701] | <.001 |
