# Supplementary material for: A Language Model for Pediatric Occupational Therapy Documentation: Model Development and Pilot Study
Source: JMIR AI. 2026 May 15;5:e73274. doi: 10.2196/73274 (PMC13179052; doi:10.2196/73274)
Supplement: Multimedia Appendix 5 [file ai-v5-e73274-s005.docx]

**Multimedia Appendix 5.** DAPT example.

The following example shows the degradation of the DAPT model compared with the original Llama 7B model. As can be seen, the DAPT note is very short and does not contain any of the contextually relevant information that the untrained Llama 2 7B chat model manages to capture despite having several errors. Text in square brackets is a description of text that is redacted to maintain privacy

**SOAP note generated by the DAPT model using the historical scratch note**

Here is a Progress Note in SOAP format.

This is a Progress Note - OT from [DATE (Incorrect)]:

Email received from [NAME] asking about [NAME] - OT responded to email today - see attached.

**SOAP note generated by the untrained Llama 2 7B chat model using the historical scratch note example**

Here is the Progress Note in SOAP format based on the Scratch Note provided:

S - Subjective:

* The child reported that the chair provided is too small for them.

* The child's occupational therapist (OT) met with the child and tried using a different chair.

* The child seemed to prefer the shorter desk height.

* The child's classroom teacher reported that the child is still comfortable with the current chair.

O - Objective:

* The child's current chair is too small for them.

* The child's desk is too tall for them.

* The child's rocker chair is appropriate and available within the school.

A - Assessment:

* The child's discomfort with the current chair may be impacting their ability to focus and participate in class.

* The child's height may be a factor in their discomfort with the chair.

P - Plan:

* Provide the child with a chair that is the appropriate size for their height.

* Lower the desk height to a level that is comfortable for the child.

* Monitor the child's comfort and participation in class.

* Follow up with the child and classroom teacher as needed.
